# The Conservative Treatment of the Dental Pulp

**Published:** 1890-09

**Authors:** A. C. Hugenschmidt

**Affiliations:** Paris, France.


ARTICLE III.

### THE CONSERVATIVE TREATMENT OF THE DENTAL PULP.

BY DR. A. C. HUGENSCHMIDT, PARIS, FRANCE.

Much has been said and written concerning pulp-capping. Many have claimed success and an equal number have reported failures. It is a noticeable fact that, though the rupture of a pulp is practically similar to a wound on other soft tissues, the treatment in vogue is usually totally different. An inflammation of the pulp is the same as any other inflamed tissues, and calls for similar treatment. The pulp is a highly organized tissue supplied with nerve, artery, and vein, and there is good reason to believe, with lymphatics as well.

When a pulp is exposed it is no uncommon practice to touch the wound with a coagulant. A surgeon who would so treat an ordinary wound would be considered insane. The coagulum, requiring adsorption, would undoubtedly act as an irritant and obstacle to the healing process. If this is true in the larger wounds of the body in tissues not over-sensitive, how much more must it be true of the dental pulp? To those who demand that a coagulum is needed as a protection to the pulp at the point of rupture, it will suffice to say that the air is a coagulant, and that blood cannot be exposed even momentarily without a delicate film being formed.

In treatment of the pulp my practice has been to wash out the cavity with sterilized water, saturated with a 1-2000 solution of bichloride of mercury. The capping is powdered iodoform, or salol mixed with lanolin and covered with mica, the rest of the cavity being filled temporarily with oxy-phosphate or oxy-chloride of zinc. Mica is a most valuable capping, being thin and a good non-conductor.

In cases where the pulp is fully exposed, unprotected by decalcified dentine, and presenting a cherry-red color in place of the normal rosy tint, it should not be capped at once. It is an inflamed wound. Where there is inflammation about a wound the surgeon hesitates about closing the opening. Even after days or weeks of treatment, when at last he concludes to unite the edges of the wound, hoping for final healing, he invariably inserts a drainage-tube.

Some of us think that all cases of pulp exposure should be capped at once. In case of inflammation the immediate capping results in inclosing inflammatory products, which become a source of irritation. Patient complains of occasional shooting pains, which may eventually pass away; but the pulp is dying. If the patient is anæmic these pains become worse till the patient insists on the removal of the filling, and the destruction of the pulp is then inevitable.

Here especially coagulants are contra-indicated. A disinfectant which is also a coagulant is self-limiting. *All below the coagulum remains in a septic condition*, the coagulum thus becoming a barrier against further treatment. Never cap at the first or second sitting. Use antiseptic dressing only. The procedure is to remove the decay, not objecting to exposing the pulp. If sensitive apply a saturate solution of muriate of cocaine, or even the crystals. The result largely depends on removing the decay, which is septic.

As a dressing iodoform powder has been my dependence since I first heard it recommended by Truman. Sometimes I use salol. Iodoform or salol powder may be used in lower teeth, but in the upper teeth it should be mixed with lanolin, applied to the exposure, and covered with mica or gold, and then with gutta-percha or oxy-phosphate. This dressing should be allowed to remain in place a week.

When removed a slight trace of pus may be detected on the absorbent cotton used to carry the dressing, in which case the dressing must be removed.

Two to four treatments should be resorted to before final capping. This final capping should only be placed when pus has ceased and the normal rosy color of the pulp has been restored. If cotton is used with gum sandarac as the temporary fillings, care must be used to separate with a piece of mica, this cotton filling, from the cotton which carries the lanolin and iodoform dressing. The permanent capping and filling has been described.

Another class of cases is where the exposed pulp is freely suppurating, and possibly neighboring parts are found in similar condition. The treatment is the same, but the dressings are to be made more frequently; they should be removed every three days. The exposure must be cauterized with carbolic acid, applied to it for a moment. The cautery is to be renewed or not as indicated by the appearance of the pulp at each dressing. If, after the fifth or sixth treatment, no improvement occurs, the case may be counted hopeless and arsenic applied.

I have met cases where the suppuration continued, though the surface of the pulp which was in sight appeared healthy. In such cases it is probable that the suppuration comes from a point on the side of the pulp near the foramen, the pus exuding between the pulp and the canal wall. In two cases having straight roots the pulps were removed under cocaine, and the microscope disclosed such a condition.

To sum up, we may say that—

First—In healthy, freshly exposed pulps salvation is not only possible but probable.

Second—Pulps long exposed but not suppurating can be saved in the majority of cases.

Third—In suppurating cases the pulp can only be saved after proper antiseptic care, and not in all cases.

Fourth—Tumors of the pulp require extirpation and the removal of the entire pulp.

DISCUSSION.

*Dr. Atkinson.*—I think that it is a very audacious statement for any man to make that he can discover that a pulp is suppurating from a point which is not visible. Any part of the body which has life enough in it to suppurate can be restored to nutrient activity and be healed by granulation, provided the neighborhood can be kept healthy.

*Dr. Ottolengui.*—I do not wish to appear to contradict or oppose Dr. Atkinson, but I wish to defend my friend Dr. Hugenschmidt. I think Dr. Atkinson has misunderstood or misapprehended him. He did not say that he can tell when a pulp is suppurating from a point not visible. He claims that in some cases where the parts appeared healthy and yet suppuration continued after five dressings, he suspected this to be the fact. He tells us that he examined two such cases after removal, and the microscope exposed such a condition.

*Dr. Atkinson.*—God pity the man who needs to dress these cases five times.

*Dr. Patrick, Illinois.*—I never have saved a ruptured pulp. I have been told that I can cut away a part of a pulp and save the rest. I can't. Atkinson says that any part of the body can be restored to health after suppuration. I say he is wrong. There are parts of the body which cannot be restored. The pulp is one; the synovial membrane is another.

*Dr. Genese.*—Would Dr. Patrick destroy a pulp exposed when healthy? I had a patient who had had a tooth filled with gold. The pulp had been destroyed and removed and the root filled; no abscess occurred. She, however, suffered much from neuralgia, and claimed that from time to time she felt something burst, after which she would have temporary relief. This "bursting" would be followed by a discharge from the nostril. As it was plain that there was pus in the antrum, the tooth was removed. The root was found necrosed. Antiseptic treatment brought about a cure. Patient has had no pain since. I relate this as a warning against the wanton destruction of pulps.

*Dr. Young, Alabama.*—Inflammation of the brain cannot be cured. A crushed limb must be cut off; it cannot be restored to health. It is the same with the exposed inflamed dental pulp. After a pulp has ached it is no longer connective tissue; a metamorphosis occurs, and it becomes striated, like muscular tissues. A painless method of applying arsenic to destroy a pulp is to use this formula: Cocaine, four parts; antipyrine, four parts; acetate of morphia, four parts; arsenic, three parts. Mix with lanolin.

*Dr. Patrick.*—No wound can heal without lymph; no lymph can be produced without the lymphatic gland, and no glands have ever been proved to exist in the pulp. I know what I am talking about. A man cannot appreciate what the use of the microscope means unless he devotes himself exclusively to it. I have spent a great deal of money and used up a great deal of time in microscopical examination of dental tissues. I say no one has shown lymphatics in a pulp.—*Dental Mirror.*

